# A cross-sectional study on the relationship between visceral adiposity index and periodontitis in different age groups

**DOI:** 10.1038/s41598-023-33082-6

**Published:** 2023-04-10

**Authors:** Qinghua Yang, Xuming Wang, Chen Li, Xuanming Wang

**Affiliations:** 1 Department of Stomatology, ShuCheng People’s Hospital, Lu’an, Anhui China; 2 Department of Stomatology, The Affiliated Huaian No. 1 People’s Hospital of Nanjing Medical University, Huaian, Jiangsu China; 3 Department of Stomatology, BinZhou Medical University, No. 346, Guanhai Road, Laishan District, Yantai City, 264003 Shandong Province China; 4 Department of Stomatology, Haiyan Stomatological Hospital, No. 89, Qinjian South Road, Haiyan County, Jiaxing City, 314399 Zhejiang Province China

**Keywords:** Diseases, Medical research, Risk factors

## Abstract

Obesity and periodontitis are significantly related, and the visceral adiposity index (VAI) is an important indicator of obesity. This study aimed to investigate the association between VAI and periodontitis. The study included participants from the 2009–2014 cycle of the National Health and Nutrition Examination Survey who received a complete periodontal exam and VAI record. Periodontitis, according to the Centers for Disease Control and Prevention-American Academy of Periodontology periodontitis case definitions, is categorized into the following: no periodontitis, moderate periodontitis, mild periodontitis, and severe periodontitis. Hierarchical analysis, multivariable logistic regression, and restricted cubic spline regression were conducted to investigate the relationship between periodontitis and VAI in adults. There was no significant relationship between VAI and the prevalence of periodontitis in all age groups (*P* = 0.08). Age-stratified analysis showed a significant association between periodontitis and VAI in adults aged 40–50 years (*P* < 0.001). After adjusting for all covariates, the association between periodontitis and VAI remained significant in the 40–50-years age group (the trend *P* value = 0.014). Restricted cubic spline analysis showed a non-linear relationship between VAI and periodontitis (*P* for non-linear = 0.002). Visceral adiposity index was significantly associated with periodontitis risk in the 40–50-year-old group, and the relationship between VAI and periodontitis risk was found to be non-linear.

## Introduction

Periodontitis is among the ten most common chronic diseases, and nearly half of the world's adults have at least one tooth with periapical periodontitis^1^. Periodontitis has now become a major public health concern and the cause of a serious economic burden on individuals^2^. The relationship between periodontitis and systemic disorders, such as diabetes, rheumatoid arthritis, cardiovascular disease, and obesity, has been the subject of contemporary studies^3^. The association between obesity and periodontitis is a novel research question in periodontal medicine, and the underlying molecular mechanisms remain unknown^4^.

It has been demonstrated that adipose tissue has different functions and that certain adipose tissues, such as brown fat, do not threaten health^5^; therefore, it cannot be presumed that high adiposity and excessive body weight are unhealthy states. Thus, studying lipid accumulation at specific locations in the body can help understand the role and importance of adipose tissue in disease pathophysiology risk prediction^6–9^.

Visceral adipose tissue can be accurately assessed by computer tomography (CT) and magnetic resonance imaging (MRI), the latter being the gold standard for its evaluation. However, this approach requires more professional equipment, therefore, cannot be used in clinical practice with large samples^10^. Thus, visceral adiposity index (VAI), based on its component indicators, waist circumference (WC), body mass index (BMI), triglycerides (TG), and high-density lipoprotein cholesterol (HDL-C), has been proposed as an indicator of fat distribution and function in the body^11^. It is considered a relatively simple, reliable, and inexpensive indicator; has been widely used to predict the risk of a variety of diseases, such as cardiovascular disease and hyperuricemia; and even has the ability to increase the risk of all-cause mortality^12,13^. Research has shown that the component indicators of VAI are significantly associated with the occurrence and development of periodontitis^14–16^.

To our knowledge, the relationship between VAI and periodontitis has not yet been explored. Therefore, we investigated this association based on national demographic data.

## Methods

### Study population

The baseline clinical data analyzed were taken from the 2009–2014 cycle of the National Health and Nutrition Examination Survey (NHANES). The present study was performed in accordance with the Declaration of Helsinki (2013 Fortaleza revision), and all methods were performed in accordance with the relevant guidelines and regulations. The data is publicly available at https://www.cdc.gov/nchs/nhanes.

**Figure 1:**
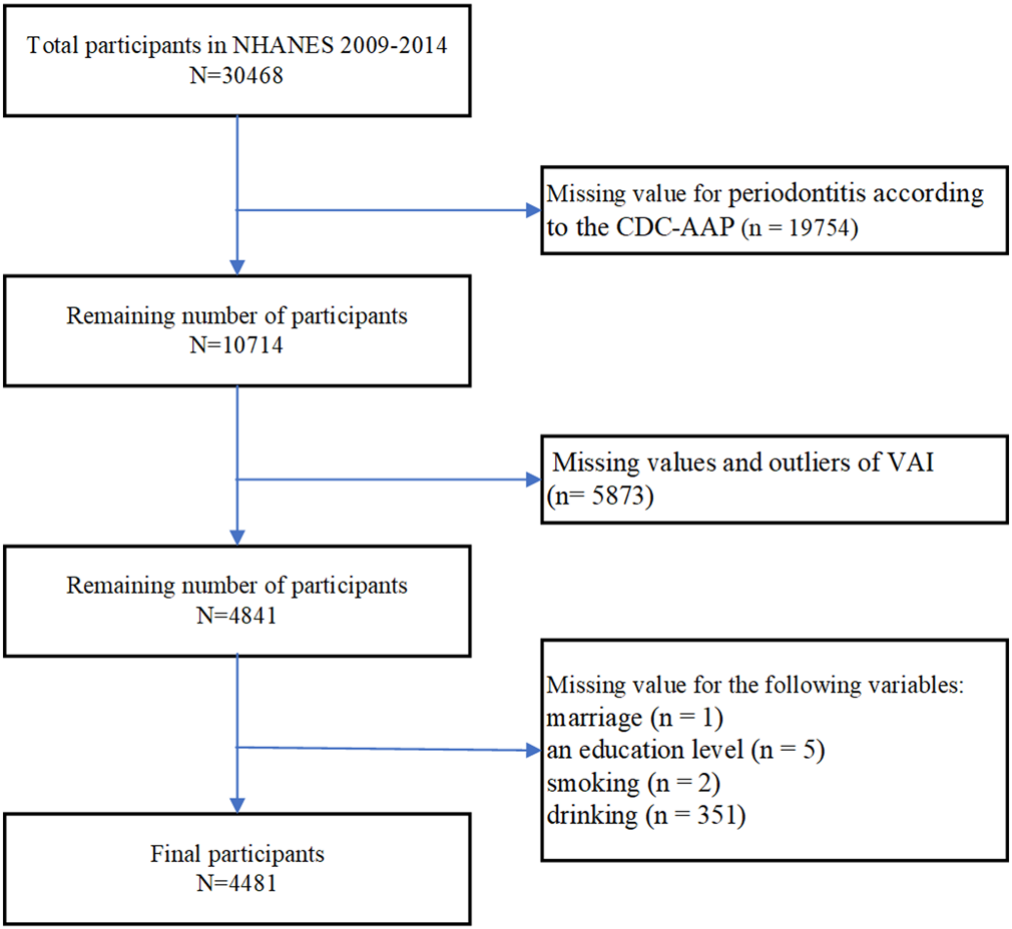
A flowchart of the participant selection process. *VAI* visceral adiposity index, *CDC-AAP* Centers for Disease Control and Prevention-American Academy of Periodontology.

There were 30,468 participants, out of which 4482 participants were eligible for inclusion in the study (Fig. 1). The participant exclusion criteria were as follows: missing value for periodontitis according to the Centers for Disease Control and Prevention-American Academy of Periodontology (CDC-AAP) periodontitis case definitions^17^ (n = 19,754), missing values and outliers for VAI (n = 5873), marriage (n = 1), an education level (n = 5), smoking (n = 2), and drinking (n = 351).

### Assessment of periodontitis

Periodontitis is divided into four categories by the CDC-AAP based on dental attachment loss and probing depth, excluding third molars: no periodontitis, mild periodontitis, moderate periodontitis, and severe periodontitis^17^. These criteria were used to classify participants. Participants in the "no" group were categorized as the "no periodontitis population," while participants in the "mild," "moderate," and "severe" groups were categorized as "periodontitis populations" in the research cohort for examining the relationship between the presence of periodontitis and VAI. Only patients with periodontitis were included in the research cohort for evaluating the relationship between the severity of periodontitis and VAI.

### Visceral adiposity index

VAI is calculated using two separate formulas for male and female participants. For males, the formula is:

$$ {\text{VAI }} = \, \left[ {{\text{WC }}\left( {{\text{cm}}} \right)/\left( {{39}.{68 } + { 1}.{88 } \times {\text{ BMI }}\left( {{\text{kg}}/{\text{m}}^{{2}} } \right)} \right)} \right] \, \times \, \left( {{\text{TG }}\left( {{\text{mmol}}/{\text{L}}} \right)/{1}.0{3}} \right) \, \times \, \left( {{1}.{31}/{\text{HDL }}\left( {{\text{mmol}}/{\text{L}}} \right)} \right) $$

For females, the formula is:

$$ {\text{VAI }} = \, [{\text{WC }}\left( {{\text{cm}}} \right)/({36}.{58 } + { 1}.{89 } \times {\text{ BMI }}({\text{kg}}/{\text{m}}^{{2}} ))] \, \times \, \left( {{\text{TG }}\left( {{\text{mmol}}/{\text{L}}} \right)/0.{81}} \right) \, \times \, \left( {{1}.{52}/{\text{HDL}}\left( {{\text{mmol}}/{\text{L}}} \right)} \right) $$

VAI was further analyzed by using quartile statistics as categorical variables.

### Covariates

BMI was calculated as the ratio of weight (kg) to height squared (m^2^). Participants were categorized into three groups: “never” (lifetime use of < 100 cigarettes), “former” (lifetime use of ≥ 100 cigarettes and no longer smoking), or “now” (lifetime use of ≥ 100 cigarettes and smoking every day or someday). Participants were categorized into similar groups based on drinking habits.

### Statistical analysis

The primary objective was to investigate whether the presence and severity of periodontitis were associated with VAI in a large-scale population survey in the United States. Complex data were calculated using descriptive statistics. Categorical variables among covariates were calculated as unweighted frequencies and weighted estimates of overall proportions, and continuous variables were presented as mean ± standard error. The chi-square test or Student's t-test was used to determine the *P* value of the distribution. Stratified analysis was used to assess the relationship between the presence and severity of periodontitis and VAI according to age. Univariate analysis and multinomial regression analyses were conducted to investigate the correlation between the presence of periodontitis and VAI. Models were adjusted for sex, ethnicity, education, smoking, and drinking. Restricted cubic spline regression was applied to assess the non-linear relationship between VAI and periodontitis. All statistical analyses in this study were carried out using the R version 4.1.2 (NHANESR package). *P* = 0.05 was used to determine the statistical significance.

### Ethics approval

The studies involving human participants were reviewed and approved by the NCHS. Institutional Review Board approval and documented consent was obtained from participants. The Research Ethics Review Committee approved the NHANES survey protocol (https://www.cdc.gov/nchs/nhanes/irba98.htm).

## Result

### Baseline characteristics of NHANES participants

The weighted characteristics of the participants are shown in Table 1. The final sample included 4482 participants, including 2255 men (50.3%) and 2227 women (49.7%). The mean age of the participants was 55.2 years, representing 61 million people in the United States. VAI ranged from 0.176 to 28.942, with a median of 1.469. All participants were categorized into four groups based on the severity of periodontitis: no, mild, moderate, and severe. The p-value of the relationship between periodontitis and VAI in all participants was 0.08. Other variables, including sex, age, race, education, marriage, smoking, alcohol consumption, WC, TG, and HDL, were found to be closely related to periodontitis (all *P* < 0.05).

**Table 1:** Demographic and clinical characteristics of study participants (n = 4482).

Variable	No	Mild	Moderate	Severe	*P* value
Age, mean (SE)	48.40 (0.43)	48.77 (0.87)	55.97 (0.50)	54.47 (0.57)	< 0.01
BMI, mean (SE)	29.16 (0.19)	30.41 (0.61)	29.45 (0.28)	29.51 (0.48)	0.21
WC, mean (SE)	99.51 (0.40)	103.54 (1.42)	102.03 (0.69)	102.87 (1.13)	< 0.01
TG, mean (SE)	1.42 (0.03)	1.76 (0.31)	1.52 (0.05)	1.60 (0.07)	0.04
HDL, mean (SE)	1.42 (0.01)	1.36 (0.03)	1.38 (0.02)	1.30 (0.02)	< 0.01
VAI, mean (SE)	1.99 (0.05)	2.66 (0.56)	2.21 (0.11)	2.19 (0.10)	0.08
Sex, N (weighted %)					< 0.01
Male	935 (44.52)	115 (53.28)	844 (55.20)	361 (76.60)	
Female	1314 (55.48)	89 (46.72)	700 (44.80)	124 (23.40)	
Ethnicity, N (weighted %)					< 0.01
Non-hispanic white	1174 (76.33)	86 (64.44)	621 (64.93)	139 (53.61)	
Non-hispanic black	339 (7.22)	44 (13.18)	331 (11.29)	152 (19.40)	
Mexican American	233 (5.50)	47 (13.31)	263 (10.67)	99 (13.09)	
Other race	503 (10.95)	27 (9.07)	329 (13.11)	95 (13.90)	
Marriage, N (weighted %)					< 0.01
Married/living with partner	1549 (74.51)	121 (62.97)	983 (65.95)	305 (59.98)	
Divorced/never married/widowed/separated	700 (25.49)	83 (37.03)	561 (34.05)	180 (40.02)	
Education, N (weighted %)					< 0.01
Less than high school	324 ( 9.05)	49 (14.69)	481 (23.67)	184 (31.83)	
High school	408 (17.22)	52 (23.52)	370 (25.00)	129 (26.65)	
More than high school	1517 (73.72)	103 (61.79)	693 (51.33)	172 (41.52)	
Smoking, N (weighted %)					< 0.01
Never	1447 (63.85)	133 (68.03)	769 (47.24)	167 (32.61)	
Former	526 (25.05)	34 (17.51)	439 (30.49)	143 (27.87)	
Now	276 (11.10)	37 (14.46)	336 (22.27)	175 (39.52)	
Drinking, N (weighted %)					< 0.01
Never	283 (9.39)	17 (6.00)	255 (12.98)	48 (7.11)	
Former	308 (12.09)	36 (14.73)	328 (18.04)	105 (19.54)	
Now	1658 (78.52)	151 (79.27)	961 (68.97)	332 (73.34)	

*BMI* body mass index, *WC* waist circumference, *TG* triglycerides, *VAI* visceral adiposity index, *HDL* high-density lipoprotein.

### Subgroup analysis

To further analyze whether there is a relationship between VAI and periodontitis, we used stratified analysis to investigate the relationship between VAI and periodontitis presence and severity by age. VAI was divided into four groups according to the quartile method for further analysis. We found a significant association between VAI and the presence of periodontitis in the 40–50-year-old group (the trend *P* value < 0.001) but not in the other age groups (Table 2). There was no significant relationship between VAI and the severity of periodontitis in all age groups (Supplementary table 1).

**Table 2:** Stratified analyses of the relationship between VAI and the presence of periodontitis according to age.

	Q1	Q2, OR (95% CI)	*P*	Q3, OR (95% CI)	*P*	Q4, OR (95% CI)	*P*	*P* for trend
Age								
30–40	Ref	1.128 (0.744,1.711)	0.563	1.132 (0.781,1.641)	0.505	1.410 (0.939,2.116)	0.096	0.103
40–50	Ref	2.018 (1.358,2.998)	< 0.001	3.162 (2.042,4.897)	< 0.0001	2.788 (1.702,4.569)	< 0.001	< 0.001
50–60	Ref	1.217 (0.798,1.857)	0.354	0.901 (0.516,1.574)	0.710	1.267 (0.748,2.146)	0.371	0.652
60–70	Ref	1.119 (0.574,2.183)	0.736	1.165 (0.670,2.026)	0.580	1.241 (0.611,2.523)	0.542	0.515
70–80	Ref	0.700 (0.477,1.025)	0.066	0.685 (0.401,1.169)	0.160	0.583 (0.313,1.088)	0.088	0.111

*VAI* visceral adiposity index

### Univariate analysis of the prevalence of periodontitis in 40–50-year-old participants

In the 40–50-years-old group, we used the results of a univariate analysis to show that the groups with the higher VAI (Q2, Q3, and Q4) had a higher prevalence of periodontitis compared to that of participants in the group with lower VAI (Q1), and the effect value and 95% CI were Q2 (2.02 (1.36, 3.00)), Q3 (3.16 (2.04, 4.90)) and Q4 (2.79 (1.70, 4.57)), respectively. Meanwhile, we found that sex, WC, BMI, TG, HDL, marriage, race, education, and smoking were correlated with the occurrence of periodontitis (all *P* < 0.05), but alcohol consumption was not (*P* > 0.05) (Table 3).

**Table 3:** Univariate analysis of variables associated with the presence of periodontitis.

Character	OR (95% CI)	*P*
Sex		
Male	Ref	Ref
Female	0.46 (0.34,0.61)	< 0.0001
BMI	1.04 (1.01,1.07)	0.003
Waist	1.03 (1.02,1.04)	< 0.0001
TG	1.24 (1.06,1.44)	0.01
HDL	0.34 (0.23,0.51)	< 0.0001
VAI		
Q1	Ref	Ref
Q2	2.02 (1.36,3.00)	< 0.001
Q3	3.16 (2.04,4.90)	< 0.0001
Q4	2.79 (1.70,4.57)	< 0.001
Ethnicity		
Non-hispanic white	Ref	Ref
Non-hispanic black	2.16 (1.39,3.35)	< 0.001
Mexican American	3.03 (2.06,4.46)	< 0.0001
Other race	1.14 (0.78,1.68)	0.48
Marriage		
Married/living with partner	Ref	Ref
Divorced/never married/widowed/separated	2.72 (1.92,3.86)	< 0.0001
Education		
Less than high school	Ref	Ref
High school	0.71 (0.46,1.09)	0.11
More than high school	0.24 (0.16,0.35)	< 0.0001
Smoking		
Never	Ref	Ref
Former	1.81 (1.15,2.85)	0.01
Now	4.09 (2.66,6.28)	< 0.0001
Drinking		
Never	Ref	Ref
Former	1.24 (0.70,2.20)	0.45
Now	0.71 (0.44,1.15)	0.16

BMI: body mass index, *WC* waist circumference, *TG* triglycerides, *VAI* visceral adiposity index, *HDL* high-density lipoprotein

### Multi-factor analysis of the relationship between VAI and periodontitis in 40–50-year-old participants

We established three logistic regression models to analyze the relationship between VAI and periodontitis among 40–50-year-old participants. In the unadjusted model, the incidence of periodontitis increased with higher VAI, and the trend *P* value < 0.001. Model 2 was adjusted according to sex, and the trend *P* value < 0.001. After adjustment for sex, race, education, and smoking, the trend *P* value = 0.014. This suggests that VAI is positively correlated with the occurrence of periodontitis, and the results are stable among 40–50-year-old participants (Table 4).

**Table 4:** Adjusted multinomial logistic regression of VAI with periodontitis in 40–50-year-old population.

	Q1	Q2, OR (95% CI)	*P*	Q3, OR (95% CI)	*P*	Q4, OR (95% CI)	*P*	*P* for trend
Model 1	Ref	2.018 (1.358, 2.998)	< 0.001	3.162 (2.042, 4.897)	< 0.0001	2.788 (1.702, 4.569)	< 0.001	< 0.001
Model 2	Ref	1.938 (1.294, 2.901)	0.002	3.178 (1.985, 5.087)	< 0.0001	2.466 (1.513, 4.020)	< 0.001	< 0.001
Model 3	Ref	1.910 (1.232, 2.961)	0.005	2.735 (1.618, 4.625)	< 0.001	2.070 (1.221, 3.511)	0.008	0.014

Model 1: Unadjusted.

Model 2: Adjusted for sex.

Model 3: Adjusted for sex, ethnicity, education, and smoking.

### The dose–response relationship between VAI and periodontitis

**Figure 2:**
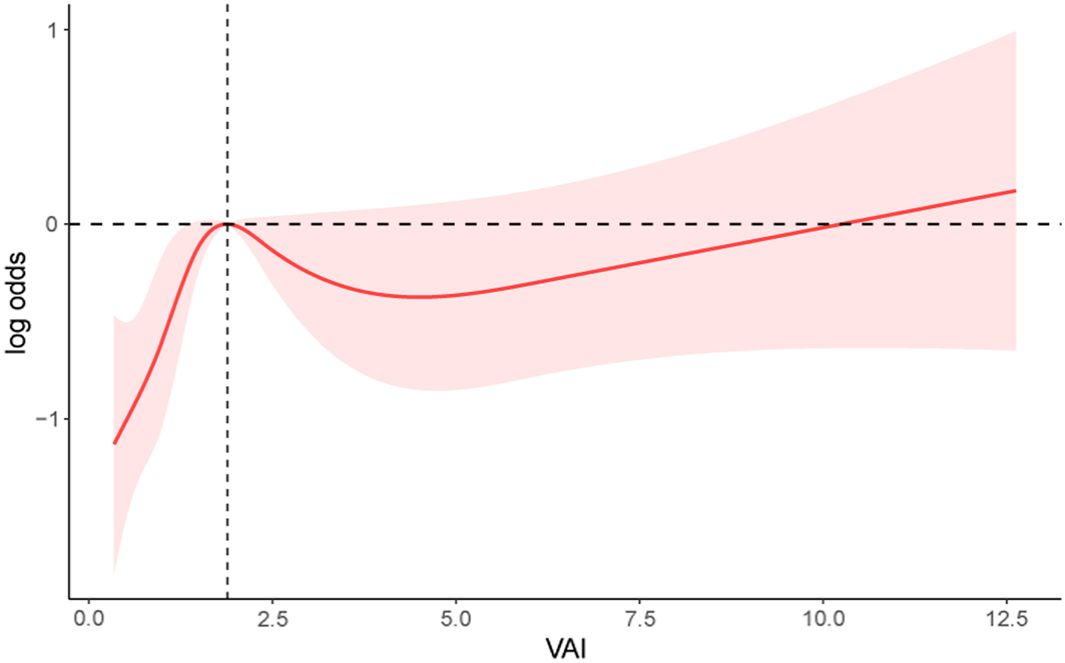
Density-dose–response correlation between the visceral adiposity index (VAI) and periodontitis. The light red area is indicated as the 95% CI. Each point illustrates the quantitative magnitude of the VAI index, which is linked into a continuous line. Adjustments were made for all individual covariates except effect modification factors. *VAI* visceral adiposity index.

Restricted cubic spline regression was applied to assess the relationship between the VAI and periodontitis. Our results showed that VAI was non-linearly related to periodontitis (*P* for non-linear = 0.002). When the VAI value is less than 1.89, the risk of periodontitis increases with the increase in VAI value; when the VAI value is between 1.89 and 4.48, the risk of periodontitis decreases with the increase in VAI value; when the VAI value is more than 4.48, the risk of periodontitis gradually increases (Fig. 2).

## Discussion

Although obesity is associated with the prevalence of periodontitis^3^, obesity indicators that can predict the risk of periodontitis remain unknown as of yet. Based on the NHANES’s large-sample database, we found that while higher VAI was associated with the prevalence of periodontitis among US adults aged 40–50 years, it was not associated with its severity. After VAI is changed from a continuous to a quadratic categorical variable, higher VAI values are significantly associated with a higher risk of periodontitis in US adults aged 40–50 years. This correlation persists after controlling for covariates, suggesting that VAI is a reliable predictor of obesity in adults with periodontitis aged 40–50 years.

A Japanese cross-sectional study by Saito et al.^18^ was the first to establish a link between obesity and periodontitis. Chaffee et al.^19^ reported that alveolar bone loss was more common in obese individuals. In addition, obese children and adolescents are more likely than non-obese individuals to have subgingival calculus and to detect gingival bleeding^20^. A recent study has found that in obese adults, excessive consumption of sweets combined with poor hygiene increases the likelihood of periodontal disease and necessitates early detection and treatment^21^. These studies showed that gum and periodontal disease are more likely to manifest in obese patients.

BMI is widely used in the assessment of obesity, but its relationship with periodontitis remains controversial^22,23^. This may be because conventional measures of obesity, such as BMI and WC, do not reflect a complete assessment of fat content, especially visceral fat content. At present, the gold standard for BMI detection is still MRI, which is time-consuming, expensive, and difficult to carry out on a large scale.

Visceral adiposity, also known as organ adiposity, is the accumulation of adipose tissue between the viscera and the trunk as opposed to subcutaneous and intermuscular fat. Kissebah et al.^24^ showed the difference between the metabolic functions of visceral and subcutaneous adiposity. Visceral adipose tissue surrounds visceral organs and communicates with them via adipokine production and secretion^25^. Visceral obesity is linked to an increase in adipocytokine production, pro-inflammatory activity, and risk of illness and mortality^26^.

VAI, as a specific indicator of visceral adiposity disorders, has recently been shown to be associated with diabetes, hypertension, hyperlipidemic pancreatitis severity, and a poor prognosis in hepatocellular carcinoma^27–30^. As mentioned before, research has shown that VAI component indicators are closely related to the occurrence and development of periodontitis^14–16^. For the first time, we examined the association between VAI and periodontitis risk and found this association only in adults aged 40–50 years. This is consistent with previous findings showing an association between obesity and periodontitis in specific age groups^31^. A further restricted cubic spline regression used to evaluate the relationship between VAI and periodontitis in 40–50-year-old adults showed a non-linear relationship. Why this correlation occurs only in this age group is a question we will investigate in future studies.

Our study has several strengths. First, we used three cycles of the NHANES database with a larger sample size. Second, weighted data estimation was used to overcome selection bias. Third, we showed the relationship between VAI and periodontitis for the first time, providing a theoretical basis for further exploration of the relationship between obesity and periodontitis. This study also has some limitations. As it was a cross-sectional study, a causal relationship could not be confirmed. In addition, this study could not covariates such as the effects of different lifestyle habits and geography that also influence the relationship between VAI and periodontitis. Therefore, we shall continue to explore these factors in depth in future studies.

## Conclusion

VAI is significantly associated with periodontitis risk in 40–50-year-old adults. The relationship between VAI and periodontitis risk in this age group is non-linear.

## Supplementary Information

Supplementary Information.

## Data Availability

The data are available from the website (https://www.cdc.gov/nchs/nhanes/index.htm).

## Abbreviations

VAI: Visceral adiposity index
HDL: High-density lipoprotein
BMI: Body mass index
WC: Waist circumference
TG: Triglycerides
NHANES: National Health and Nutrition Examination Survey
CDC-AAP: Centers for Disease Control and Prevention-American Academy of Periodontology
