# Supplementary material for: A cross-sectional study on the relationship between visceral adiposity index and periodontitis in different age groups
Source: Sci Rep. 2023 Apr 10;13:5839. doi: 10.1038/s41598-023-33082-6 (PMC10086006; doi:10.1038/s41598-023-33082-6)
Supplement: Supplementary file 1 — Supplementary Information. [file 41598_2023_33082_MOESM1_ESM.docx]

Supplementary table 1. Stratified analyses of the relationship between VAI and the severity of periodontitis according to age

|  | Q1 | Q2, OR(95%CI) | *P* | Q3, OR(95%CI) | *P* | Q4, OR(95%CI) | *P* | *P* for trend |
| --- | --- | --- | --- | --- | --- | --- | --- | --- |
| Age |  |  |  |  |  |  |  |  |
| 30-40 | ref | 1.429(0.618,3.306) | 0.395 | 0.809(0.306,2.138) | 0.663 | 1.341(0.422,4.262) | 0.612 | 0.83 |
| 40-50 | ref | 0.578(0.257,1.301) | 0.180 | 0.691(0.271,1.759) | 0.429 | 1.060(0.475,2.365) | 0.884 | 0.394 |
| 50-60 | ref | 1.367(0.780,2.397) | 0.267 | 1.418(0.640,3.139) | 0.381 | 1.167(0.446,3.052) | 0.748 | 0.809 |
| 60-70 | ref | 1.223(0.403,3.707) | 0.716 | 0.896(0.403,1.992) | 0.783 | 1.021(0.423,2.466) | 0.962 | 0.882 |
| 70-80 | ref | 1.335(0.532,3.348) | 0.529 | 0.798(0.319,1.997) | 0.622 | 0.546(0.213,1.401) | 0.201 | 0.115 |

**Abbreviations:** VAI: Visceral Adiposity Index;
